# Chemotherapy payload of anti-insoluble fibrin antibody-drug conjugate is released specifically upon binding to fibrin

**DOI:** 10.1038/s41598-018-32601-0

**Published:** 2018-09-21

**Authors:** Hirobumi Fuchigami, Shino Manabe, Masahiro Yasunaga, Yasuhiro Matsumura

**Affiliations:** 1 Division of Developmental Therapeutics, Exploratory Oncology Research & Clinical Trial Centre, National Cancer Centre, 6-5-1 Kashiwanoha, Kashiwa, Chiba, 277-8577 Japan; 2 Department of Integrated Bioscience, Graduate School of Frontier Sciences, The University of Tokyo, 5-1-5 Kashiwanoha, Kashiwa, Chiba, 277-8561 Japan; 3 Synthetic Cellular Chemistry Laboratory, RIKEN, 2-1 Hirosawa, Wako, Saitama, 351-0198 Japan

## Abstract

Cancer-induced blood coagulation in human tumour generates insoluble fibrin (IF)-rich cancer stroma in which uneven monoclonal antibody (mAb) distribution reduce the potential effectiveness of mAb-mediated treatments. Previously, we developed a mAb that reacts only with IF and not with fibrinogen (FNG) or the fibrin degradation product (FDP). Although IF, FNG and FDP share same amino acid sequences, the mAb is hardly neutralised by FNG and FDP in circulation and accumulates in fibrin clots within tumour tissue. Here, we created an antibody drug conjugate (ADC) using the anti-IF mAb conjugated with a chemotherapy payload (IF-ADC). The conjugate contains a linker severed specifically by plasmin (PLM), which is activated only on binding to IF. Imaging mass spectrometry showed the substantial intratumour distribution of the payload following the IF-ADC injection into mice bearing IF-rich 5–11 xenografts derived from pancreatic tumours of *LSL-Kras*^G12D/+^; *LSL-Trp53*^R172H/+^; *Ptf1a-Cre* (KPC) mice. IF-ADC treatment significantly extended the survival of the KPC mice. These data suggest that conjugating chemotherapy drugs to this IF-specific mAb could represent an effective means of treating stroma-rich tumours

## Introduction

We previously reported that a “malignant cycle of blood coagulation” generates versatile cancer stroma consisting of cancer invasion into vessels, hemorrhage, insoluble fibrin (IF) formation, and replacement of the IF with collagenous tissue^1–3^, especially in invasive cancers such as pancreatic cancer, stomach cancer, glioblastoma and others. Platelet aggregation also occurs at the site of cancer-induced injury. Cancer-induced blood coagulation involves both intrinsic and extrinsic blood coagulation. The intrinsic blood coagulation in the tumour tissue produced several bradykinins^4^, and a vascular endothelial growth factor (VEGF) is known to be produced at the site of extrinsic blood coagulation^5^. Both bradykinin and VEGF are vascular permeability factors, and they can cause several inflammation events followed by the accumulation of cancer-associated fibroblasts and other cells^6–9^. Consequently, cancer induced blood coagulation generates IF rich tumour stroma^10^.

To minimise the toxic effects of anticancer agents (ACAs) on healthy human cells, drug delivery systems (DDSs) must be developed that target chemotherapeutic agents directly to cancer tissues. DDSs have been developed using the concept of the enhanced permeability and retention (EPR) effect^11,12^. Based on the EPR effect, various formulations of ACA- and gene-delivery systems have been produced and introduced in the clinic^13,14^. However, the EPR effect has not been recognised well in clinics. Above all, therapy using a DDS is not a mainstream in oncology. For example, MCC-465, categorised by a DDS consisting of a doxorubicin-incorporating liposome conjugated with polyethylene glycol (PEG) and an anti-GAH antibody that specifically binds to stomach cancer cells, was developed^15^. The formulation was highly anticipated in the field of oncology because the formulation can utilise the EPR effect, and it is simultaneously equipped with active targeting due to the anti-GAH antibody. In fact, animal experiments revealed a remarkable antitumour effect on 2 kinds of stomach cancer xenograft models^15^. Unexpectedly, in the clinical trials for patients with stomach cancer, no antitumour response was observed^16^.

It is well known that clinical pancreatic cancer tissues possess abundant cancer stroma^17,18^. On the other hand, only tumour cells occupied the pancreatic tumour xenografts; there was little tumour stroma (Supplementary Fig. 1a). From these results, we concluded that DDSs are effective in experimental pancreatic tumour xenografts because of the lack of tumour stroma. This may allow DDSs to distribute in the whole tumour tissue. On the other hand, DDSs are not effective in clinical human pancreatic cancer because of the stromal barrier that prevents the DDSs from reaching cancer cells.

IF is produced from fibrinogen (FNG) in the tumour tissue (Supplementary Fig. 2a), and the IF subsequently degraded via the fibrinolysis mechanism by plasmin (PLM) that is produced only in the presence of IF in the tumour tissue (Supplementary Fig. 2b). The fibrin degradation products (FDP) easily dissolved in the circulating blood. Therefore, IF only exists in pathological conditions, including cancer^1,19^. We previously showed that IF deposition in non-malignant diseases such as acute infarction, arthritis, and trauma occurred only at the onset or during their active phase. Subsequently, IF deposition disappeared as a result of PLM digestion and collagen replacement after a few weeks^19^. It should be noted that IF deposition in non-malignant diseases is inevitably accompanied by some symptoms related to the condition. On the other hand, IF deposition in the tumour is not associated with any symptoms. We concluded that asymptomatic and continuous IF deposition is specific to cancer^1^. We regarded IF as a potential target for cancer therapy in stroma-rich tumour such as pancreatic cancer. While IF deposition occurs in some non-malignant disorders, it is always associated with disease symptoms in these cases. Therefore, we propose that asymptomatic IF deposition is characteristic of cancer. We therefore tried to make a monoclonal antibody (mAb) against IF. We succeeded in developing a mAb that reacted only with human IF and not with human FNG and FDP^2,19^. Our anti-IF mAb recognises the unexplored pit structure of IF that is formed by conformational change of the FNG when it converted to the IF^19^. The fundamental point is that the anti-IF mAb is not neutralised by FNG and FDP in the blood despite FNG, IF and FDP are originated from same gene and share the same amino acid sequences.

In this study, we developed an antibody-drug conjugate (ADC) using the anti-IF mAb conjugated with MMAE via PLM cleavable linker because PLM is known to be converted from its inactive zymogen form plasminogen (PLG) by the action of plasminogen activator (PA) in the presence of IF. The ADC could be tested in a meaningful tumour model possessing stroma with IF. The goal of this study was to determine whether conjugating an anticancer drug to the anti-IF mAb could improve its effectiveness compared to untargeted administration of the same drug and we emphasise the limitation of xenografts model used worldwide.

## Results

### Synthesis and physical properties of antibody-drug conjugate

**Figure 1:**
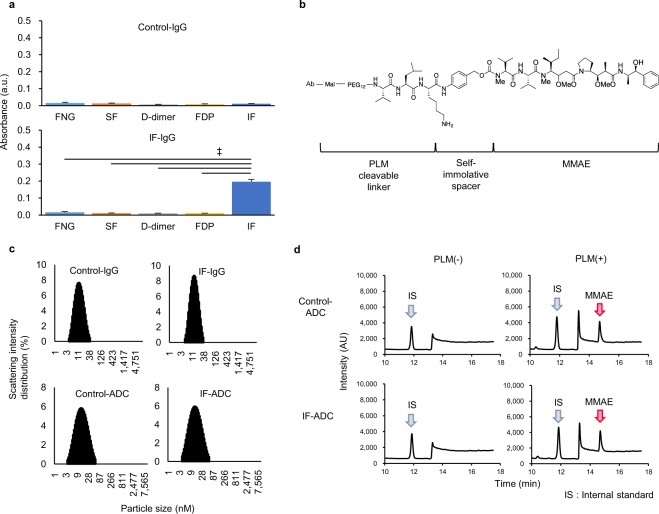
Characterization of anti-IF mAb and IF-ADC. (**a**) Specificity of anti-IF IgG. Reactivities of control antibody and anti-IF antibody were estimated against fibrinogen (FNG), soluble fibrin (SF), D-dimer, fibrin degradation product (FDP) and IF by ELISA (n = 3). Anti-IF mAb specifically recognised IF, but did not recognised its zymogen FNG and its degradation products FDP and D-dimer, whereas control mAb did not recognised FNG, IF, FDP and D-dimer. The results are shown as means ± standard deviation (s.d.). Statistical analysis was performed using the Dunnett test. ^‡^P < 0.01. (**b**) Structure of antibody-drug conjugate. MMAE was conjugated to mAb via PLM cleavable linker and self-immolative spacer. (**c**) Particle size of mAbs and ADCs. The molecular sizes of the ADCs did not change as compared to that of the corresponding antibody. This may implicate that ADCs were not fragmented or aggregated. (**d**) Chromatography of released MMAE. The peak at a retention time of 11.8 min is the ibuprofen (IS) peak (blue arrow), and the peak at a retention time of 14.7 min indicates MMAE (red arrow). Control- and IF-ADC released MMAE only when PLM was added, but there was virtually no MMAE release in the absence of PLM.

Anti-IF mAb or a control IgG was established and Anti-IF specifically recognised IF, but did not recognised its zymogen FNG and its degradation products FDP and D-dimer, whereas control mAb did not recognised FNG, IF, FDP and D-dimer (Fig. 1a). To test whether the anti-IF mAb could be used to target a chemotherapeutic agent to a tumour, we created ADCs using the anti-IF mAb (IF-ADC) or a control IgG (Control-ADC) conjugated with the anticancer agent monomethyl auristatin E (MMAE) via a Val-Leu-Lys linker. The linker is specifically severed by PLM^20^, because PLM is activated exclusively on the IF and it is usually maintained as an inactive zymogen in the blood^21^ (Fig. 1b). The molecular sizes of the ADCs did not change as compared to that of the corresponding antibody (Fig. 1c, Supplementary Table 1a). This may implicate that ADCs were not fragmented or aggregated. One molecule of the mAb was conjugated with three molecules of MMAE according to Ellman assay^22^.

### Stability of ADC and plasmin-mediated release of payload

To test whether the ADC generated as described above releases its payload in a PLM-dependent manner, we quantified MMAE by HPLC. In Dulbecco’s phosphate-buffered saline (Vehicle), Control- and IF-ADC released MMAE only when PLM was added, but there was virtually no MMAE release in the absence of PLM (Fig. 1d and Supplementary Table 1b), suggesting that the linker was cleaved by PLM and did not cleaved spontaneously in vehicle at least during the incubation time, as expected.

**Figure 2:**
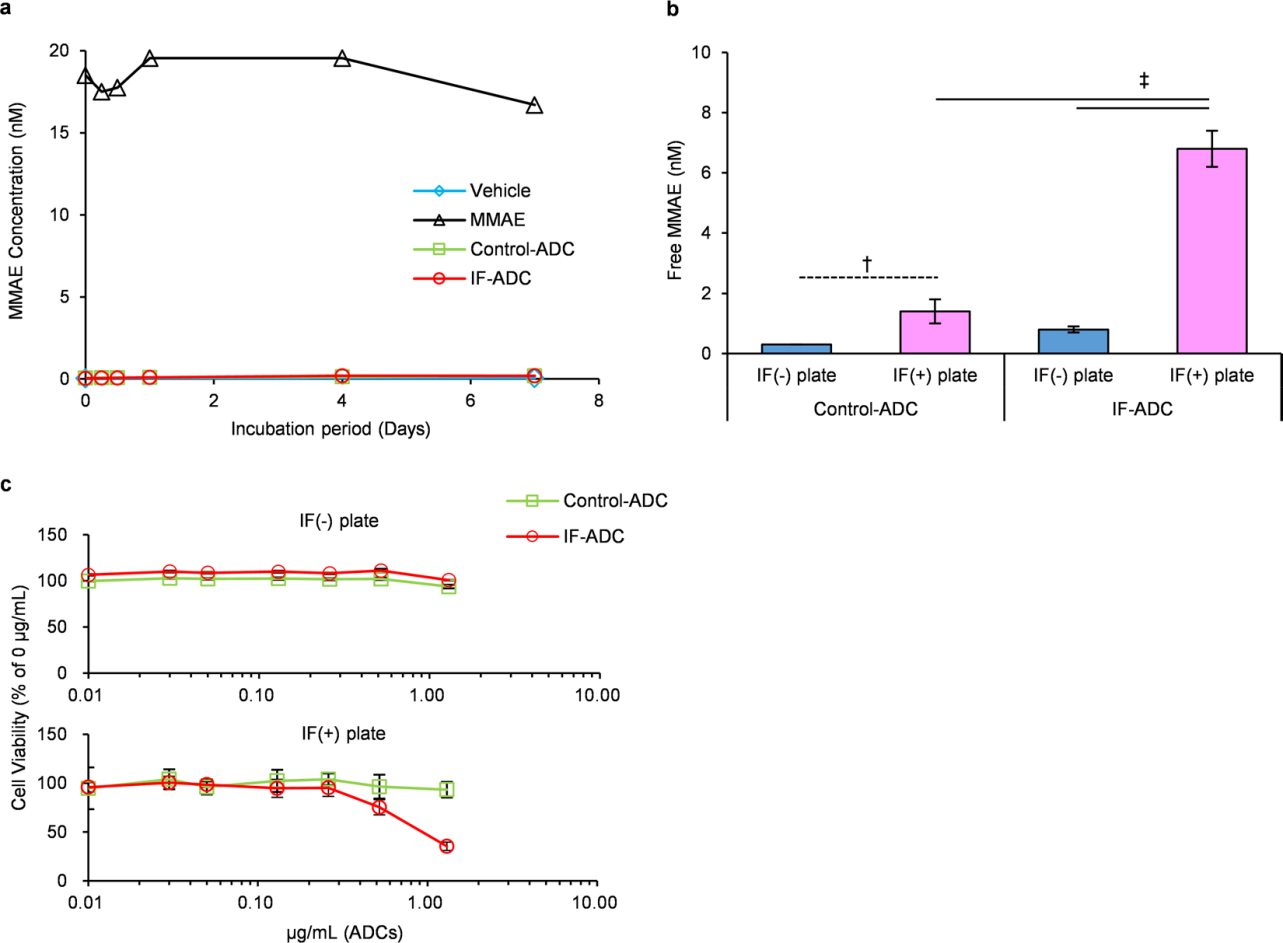
Stability in plasma and *in vitro* cytotoxicity of ADCs. (**a**) Stability of ADCs in human plasma. MMAE and ADCs were incubated in plasma for 0–7 days, and free MMAE in plasma was measured (n = 1). MMAE release from both ADCs appeared to be below the lower limit of quantitation for 7 days. (**b**) MMAE release from ADCs in the absence/presence of IF. Each ADC was incubated in the absence (IF(−)) or presence (IF(+)) of IF with 0.03 nM PA, 150 nM PNG and 100 nM α_2_-AP (n = 3). IF-ADC released significantly higher amounts of MMAE on IF-coated plates compared to Control-ADC. Statistical analysis was performed using the Dunnett test. ^†^P < 0.05. ^‡^P < 0.01. (**c**) *In vitro* cytotoxicity of ADCs. Two thousand 5–11 cells were incubated with 0–1.25 mg/mL ADCs and media containing 0.3 nM PA, 1.5 µM PNG and 1.0 µM α_2_-AP in the absence/presence of IF (n = 3). IF-ADC was only cytotoxic for 5–11 cells on the IF-coated plate whereas Control-ADC did not exhibit cytotoxicity regardless of the presence or absence of IF.

The stability of ADCs in the plasma was next evaluated, and MMAE release from both ADCs appeared to be below the lower limit of quantitation for 7 days (Fig. 2a, Supplementary Table 2a). Seven days after the incubation in plasma, PLM was added 2 h before the end of incubation. Released MMAE was then measured. The results showed that Control- and IF-ADC could completely release MMAE after the addition of PLM to the plasma (Supplementary Fig. 3). LC/MS/MS results indicated that both Control- and IF-ADC were stable in plasma after at least 7 days of incubation, and the linker sequence was cut efficiently by a certain amount of PLM in plasma. These results may indicate that ADCs were almost stable in the IF-absence plasma, and retained release ability of MMAE at least after 7 days incubation in plasma.

### Selective release of MMAE in the presence of IF

To test whether the ADC is cleaved by PLM specifically in the presence of IF, we measured the release of MMAE from the IF-ADC on IF-coated plates in the presence of PA, PLG and α_2_-antiplasmin (AP), an innate plasmin inhibitor in the body. Concentration of PA, PLG, and α_2_-AP were 150 nM, 0.03 nM and 100 nM, respectively. These levels are equivalent to normal physiological conditions. Small amounts of MMAE were released from Control-ADC on IF-coated plates compared to IF-free plates. This is probably because PLG was converted to PLM on the IF plate, and the activated PLM cut the linker to release MMAE from the Control-ADC in the closed system on the plate (Fig. 2b, Supplementary Table 2c). As expected, even in the presence of α_2_-AP, IF-ADC released significantly higher amounts of MMAE on IF-coated plates compared to Control-ADC (Fig. 2b). Taken together, these results suggest that effective sustained release of free MMAE may occur when IF-ADC binds to the IF.

### Cytotoxicity of ADC in cell lines and mouse xenograft models

In order to test the *in vitro* cytotoxicity and *in vivo* antitumour effect, we developed a 5–11 cell line from pancreatic tumour in *LSL-Kras*^*G12D/+*^; *LSL-Trp53*^*R172H/+*^; *Ptf1a-Cre* (KPC) mice. It was found that 5–11 cell can make subcutaneous tumour in nude mice and the tumours possess fibrin-rich tumour stroma (Supplementary Fig. 1b). Cells were cultured with either Control- or IF-ADC in the presence of PA, PLG and α_2_-AP in non-coated or IF-coated plates. IF-ADC was only cytotoxic for 5–11 cells on the IF-coated plate as shown in human pancreatic cancer cell line (Supplementary Fig. 5). This cytotoxicity was observed even in the presence of α_2_-AP. Control-ADC did not exhibit cytotoxicity regardless of the presence or absence of IF (Fig. 2c). The IC_50_ values of IF-ADC for several human pancreatic cell lines ranged from 0.16 to 1.19 µg/mL (Supplementary Fig. 5). The IC_50_ value of IF-ADC for 5–11 cell line was 0.93 µg/mL.

**Figure 3:**
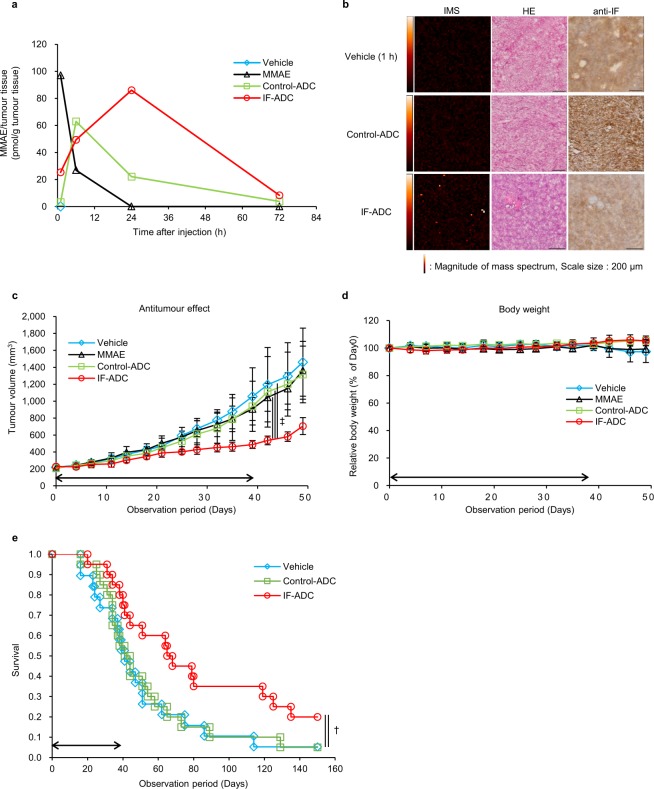
Antitumour effect and time course of MMAE in the tumours delivered by ADCs. (**a**) Time course of free MMAE in a tumour measured by LC-MS/MS (n = 1). (**b**) Imaging mass spectrometry of free MMAE in tumour specimens of ADCs after 24 h of administration (Vehicle; 1 h). Left panel shows IMS, middle panel shows HE staining, and right panel shows anti-IF staining of each specimen. Colour dots from red to white denote drug signal originating from MMAE. (**c**) Antitumour effect in mice bearing 5–11 xenografts. MMAE and ADC dosage and observation were started when the tumour volume reached approximately 200–300 mm^3^ with 20 mg/kg ADC (equivalent to 0.3 mg/kg MMAE) or 0.3 mg/kg MMAE twice a week, 12 times total intravenously. Vehicle was administered as a control at equal volume of ADCs. (n = 7). Statistical analysis was performed using one-way repeated measurement ANOVA. ^‡^P < 0.01. (**d**) Body weight change in mice bearing 5–11 xenografts. Body weight was measured before every injection or once a week after the administration period. (**e**) Survival of KPC mice over time with and without ADC treatment. Eight-week-old KPC mice began being administered ADCs with 20 mg/kg (equivalent to 0.3 mg/kg MMAE) twice a week, 12 times total intravenously. Vehicle was administered as a control at equal volume of ADCs. All results are presented as the mean ± s.d. Survival curves were described with the Kaplan-Meier method (n = 20), and comparison of survival curves was carried out with the Log-rank test. ^†^P < 0.05.

*In vivo* antitumour effects were assessed on mouse xenograft models generated using 5–11 pancreatic tumour cells. Athymic nude mice bearing 5–11 tumours were intravenously given 20 mg/kg (0.3 mg/kg MMAE equivalent) IF-ADC, Control-ADC, 0.3 mg/kg MMAE or Vehicle. IF-ADC significantly suppressed tumour growth compared to Vehicle, MMAE and Control-ADC (Fig. 3c). The percentage of tumor cells expressing Ki67 was determined by immunohistochemical method and calculated as percentage of stained nuclei. The percentage of Ki67 positive cells of Vehicle, Control-ADC and IF-ADC were 23.5, 50.5 and 11.7, respectively (Supplementary Fig. 6 and Supplementary Table 4). The body weight did not change in any experimental groups (Fig. 3d). These results suggest that effective sustained release of free MMAE may occur when IF-ADC binds to the IF in the body resulting in suppressing tumour growth in xenograft model.

### Release and distribution of intratumoural free MMAE

In order to evaluate the efficiency of MMAE release from ADCs within tumour tissues quantitatively and visually, we injected intravenously Control- and IF-ADC into mice bearing 5–11 xenografts and conducted pharmacological studies of ADCs using LC/MS/MS and imaging mass spectrometry (IMS). LC-MS/MS data showed that the area under the curve (AUC) of the released MMAE in tumours of the IF-ADC group increased compared to the Control-ADC and MMAE alone groups (Fig. 3a and Supplementary Table 3).

Unlike conventional tools such as HPLC and LC/MS, our previous study showed that IMS enables us to visualise free MMAE released from the ADC and not MMAE conjugated to mAb on the tumour tissue section^23^. Therefore, to evaluate the distribution of MMAE within tumours, target tumours were resected at 24 h after the injection of Control- and IF-ADC and analysed by IMS. Substantial amounts of free MMAE were detected after the injection of IF-ADC compared to Control-ADC at the IF-rich stroma region, suggesting that MMAE actually released in the tumour after the 24 h of injection (Fig. 3b). As long as IF exists within the tumour tissue, PLM continues to be activated on the IF and effective sustained release of free MMAE may occur.

### Survival of KPC mice with and without ADC treatment

Finally, we evaluated the antitumour effect of the ADC in KPC mice, which are a genetically engineered pancreatic tumour model. The tumours appeared to have remarkable IF deposition and abundant tumour stroma, similar to human pancreatic cancers^24^ (Supplementary Fig. 1a, b). Mice were intravenously given 20 mg/kg (0.3 mg/kg MMAE equality) ADCs or Vehicle. Kaplan-Meier curves show that the IF-ADC group exhibited significantly extended survival of the KPC mice compared to the Vehicle and Control-ADC groups (Fig. 3e).

## Discussion

In this study, we succeeded in incorporating MMAE into the anti-IF mAb via linkage with a peptide specifically recognised by PLM. PLM is produced only on the IF^24^ in tumour tissues. Biochemical studies showed that one molecule of the mAb appeared to be conjugated with three molecules of MMAE. The ADCs were stable in vehicle, and they were cut by PLM to release MMAE efficiently. This was completely inhibited by α_2_-AP, which is an innate PLM inhibitor in the body. On the IF plate, MMAE release was more significant in the presence of PLG and PA compared to the Control-ADC. From the *in vitro* results on the IF plate, IF-ADC only killed cells on IF-plate in culture even in the presence of α_2_-AP. However, as expected, Control-ADC did not. It is well known that PLM is converted from its inactive zymogen form PLG, by the action of PA in the presence of IF^21^.

As described earlier, one of the most pivotal issues in this study was to use the *in vivo* tumour model possessing abundant tumour stroma with IF. We adopted genetically engineered KPC mice developed by Tuveson *et al*., because the spontaneous pancreatic tumour was known to possess abundant tumour stroma, as in human pancreatic ductal adenocarcinoma^25^. We found that there was remarkable IF deposition within the tumour^26^ (Supplementary Fig. 1b). In this study, we succeeded in obtaining KPC mice by stably using *in vivo* fertilization according to Iwamatsu and Chang^27^, and embryo transfer according to Nakagata^28^. We could therefore conduct *in vivo* antitumour evaluation using KPC mice. We also succeeded in establishing tumour cell lines from the spontaneous tumour in the KPC mice. Inoculated tumours in nude mice appeared to possess abundant tumour stroma with IF deposition (Supplementary Fig. 1b).

The feasibility of the IF-ADC was evaluated using spontaneous tumour of the KPC mouse and 5–11 xenograft model with IF deposition. We demonstrated for the first time that the anti-IF mAb conjugated with MMAE via a plasmin cleavable linker showed that there is more MMAE at the tumour site. Furthermore, it exerted a significant antitumour effect against stroma-rich xenograft model. According to previous data^11,14^, we anticipated that Control- and IF-ADC selectively accumulate in tumour tissues via the EPR effect. As a result of the study using the IMS spectrometer, we hypothesise that only IF-ADC could release MMAE selectively after getting into the epitope domain on the IF. The increased release of drug from IF-ADC occurred because of PLM-induced cleavage of the ADC as a result of its proximity to IF, which was achieved thanks to the IF-targeting antibody. Correspondingly, IF-ADC had a stronger antitumour effect in a mouse pancreatic cancer xenograft model than the Control-ADC and MMAE alone. In addition, the IF-ADC significantly improved survival compared to vehicle and Control-ADC in the genetically engineered KPC mice bearing spontaneous pancreatic tumours. It is anticipated that the release of the drug only at the site of IF, and PLM’s inactivation by α_2_-AP elsewhere in the body, ensures that the cytotoxic effects are only targeted at the IF-regions of the tumour.

The safety issue should also be considered for therapeutic use in clinical practice. Our previous study clarified that neither IF gel formation nor degradation was affected^26^. These previous data indicate that our anti-IF mAb may not cause thrombosis or bleeding as a side effect, although these should be clarified more precisely in terms of biochemical test and pathological study in future. We previously reported that the epitope sequence of the anti-IF mAb was highly conserved in mammals, and we confirmed that our anti-IF mAb cross-reacted with mouse and human IF clots^19^. Therefore, the results of this study may be reproduced in humans.

**Figure 4:**
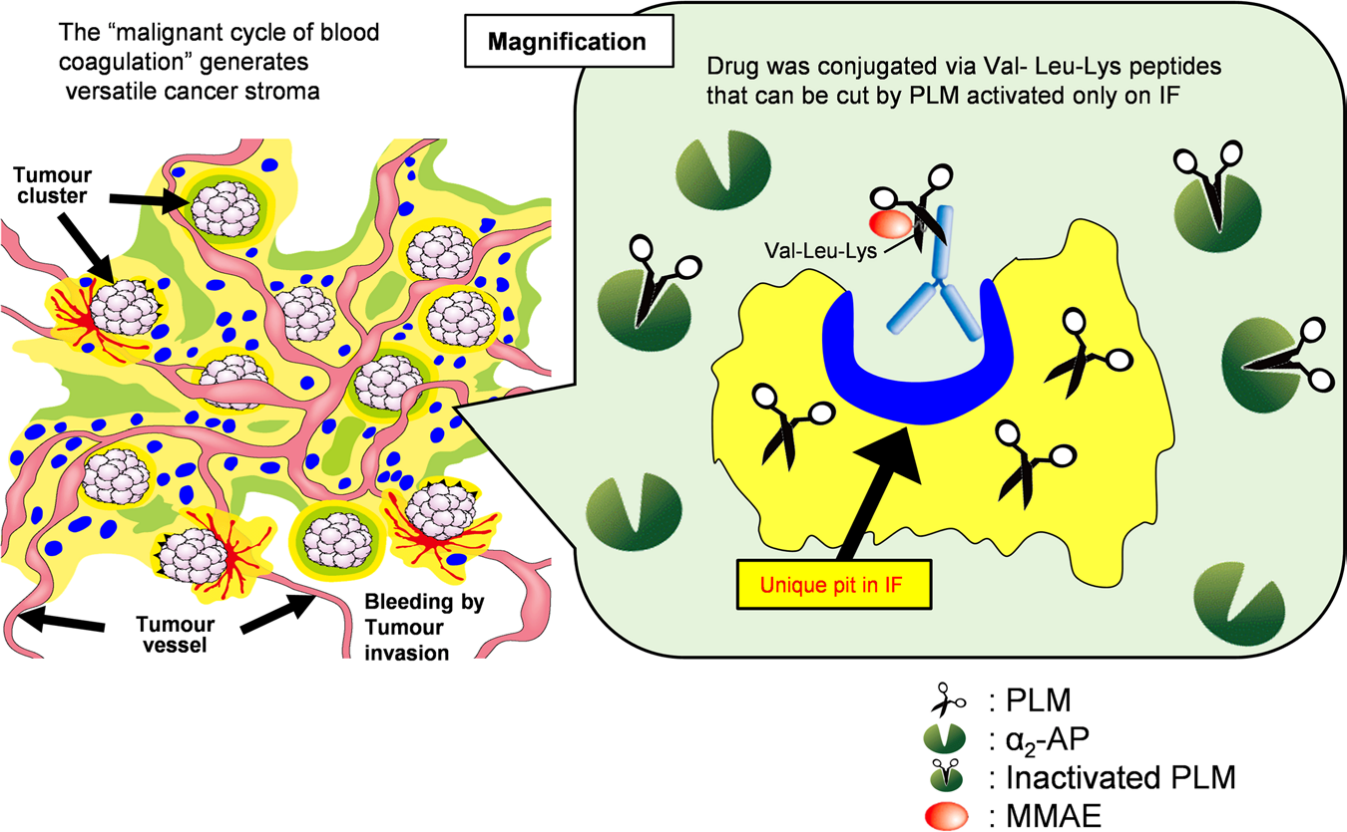
Concept of cancer stromal targeting (CAST) therapy. IF-ADC may selectively extravasate due to leaky tumour vessels, bind to the specific pits in the fibrin clot that are uncovered only when a fibrin clot forms, and create a scaffold from which effective sustained release of the free MMAE may occur only from the IF-ADC bound to IF after cutting the Val-Leu-Lys linker susceptible to PLM because PLM is known to be produced exclusively on the IF from PLG by PA and to be neutralised by α_2_-AP other than IF. Free MMAE may easily reach cancer cells by diffusing through the stromal barrier. MMAE released from IF-ADC may also attack the tumour vascular endothelial cells.

Our strategic concept of cancer stromal targeting (CAST) therapy is that the IF-ADC may selectively extravasate due to leaky tumour vessels. It can then access and bind to the specific pits in the fibrin clot that are uncovered only when a fibrin clot forms. This creates a scaffold from which effective sustained release of free ACA may occur, as a result of cleavage of the Val-Leu-Lys linker by PLM. PLM is known to be produced exclusively on fibrin clots from PLG by PA and to be neutralised by α_2_-AP in the blood stream. Free ACA may therefore easily reach cancer cells by diffusing through the stromal barrier. ACA released from IF-ADC may also attack the tumour vascular endothelial cells (Fig. 4). Furthermore, we will have to see the effect of the IF-ADC on the immunological change within the tumour tissue because ACA is released from ADC on fibrin clot in the tumor stroma.

This study indicates that CAST therapy may have promise as a new oncological modality for invasive cancers characterised by abundant tumour stroma with fibrin clots. This strategic concept should be verified by IF-ADC progress into the clinic.

## Methods

### Antibody preparation

IF-specific epitope peptides and Tag peptides were synthesized and used to immunise mice. Spleen cells from immunised mice were fused with myeloma cells (P3X63Ag8.653) as previously described^19^. Anti-IF mAb-producing hybridoma clone 99–5 and anti-Tag (Control) mAb-producing hybridoma clone 2545 were established as previously described^3^. Both hybridomas were cultured with Hybridoma–SFM (Gibco)-containing supplement. Antibodies were obtained from culture supernatant by using Protein G sepharose (GE, USA) followed by size exclusion chromatography using Superdex 200 pg (GE, USA).

### Enzyme-linked immunosorbent assay (ELISA)

IF mAb and Control mAb were conjugated with horseradish peroxidase using the Peroxidase Labeling Kit–NH_2_ (Dojindo Molecular Technologies, Japan) and diluted with Tris-buffered saline (TBS) at 1 µg/mL. Specific binding to IF, but not to FNG, SF, and FDP, was confirmed by ELISA as previously described^19^.

### Linker preparation

MMAE was purchased from Medchem Express (USA). Mal-PEG_12_-NHS was obtained from IRIS Biotech GMBH (Germany). Fmoc–amino acids were purchased from Watanabe Chemical Industries (Japan). Fmoc-Val-Leu-Lys(Mmt)-OPAB was prepared by conventional peptide synthesis from Fmoc-Lys(Mmt)-OPAB. MMAE was added via Fmoc-Val-Leu-Lys(Mmt)-OPAB-PNP. After Fmoc removal under basic conditions, Mal-PEG_12_-OSu was reacted to give Mal-PEG_12_-Val-Leu-Lys(Mmt)-OPAB-MMAE. Finally, Mmt was moved under 5% TFA-CH_2_Cl_2_ in the presence of MeOH. (PAB = *p*-aminobenzyl; PNP = *p*-nitrophenyl carbonate; Mmt = 4-methyl trityl) (Supplementary Fig. 4).

### Conjugation

The antibodies were reduced with 7.5 mM cysteamine hydrochloride (Sigma, USA) and incubated for 30 min at 37 °C. The reaction solution was immediately removed with ultrafiltration (NMWL 30 kDa). Free thiols were determined with an Ellman assay^22^ using solution of 200 µM 5,5′-dithiobis(2-nitrobenzoic acid) (Sigma). Reduced antibodies were mixed with the Mal-PEG_12_-Val-Leu-Lys-MMAE, which was prepared to a 2-fold concentration of free thiols and incubated overnight at 4 °C. Unreacted linker was removed by ultrafiltration (NMWL 30 kDa), and the buffer was exchanged with Dulbecco’s phosphate buffered saline (Sigma, USA). ADCs were checked with an Ellman assay^22^, and unreacted thiols and the drug-antibody ratio were determined.

### Particle size of ADCs

Solutions of 1 mg/mL mAb and ADCs were used to measure their particle size with a Delsa™ nano (Beckman coulter, USA) in the particle size mode.

### Release test by high-performance liquid chromatography (HPLC)

MMAE (10 µM) and ADCs (equivalent to 10 µM MMAE) were prepared. To Control- and IF-ADC solution, 1.5 µM PLM was added and incubated for 2 h at 37 °C to see how much MMAE could be released from each ADC. Protein residue was removed by acetone precipitation. The supernatant was dried and solidified using a centrifugal concentrator (Tomy, Japan). Free MMAE from ADCs was measured using the Nexera X2 HPLC system (Shimadzu, Japan). Disodium phosphate (10 mM, pH 6.8) was used for mobile phase A, and acetonitrile was used for mobile phase B. Samples were separated using the CAPCELL PAK C18AQ column (3 μm polar, 4.6 mm i.d. × 100 mm, Shiseido) heated to 40 °C and equilibrated with 10% mobile phase A. MMAE was eluted using 52% mobile phase A and detected using detection wavelength 224 nm. Ibuprofen (Sigma) was used for an internal control, was eluted by 27% mobile phase A and was detected at the same detection wavelength as MMAE.

### Cell culture

From pancreatic cancers in KPC mice, 5–11 cells were established as previously described^29^. Then KRAS and Trp53 mutation were confirmed by direct sequencing (Supplementary Fig. 1c, Supplementary Table 1c). These cells were maintained with RPMI containing 10% fetal bovine serum and transplanted into animals within 5 passages after established. Cells were not used beyond 7 passages after establishment because we found that xenograft tumour kept abundant stroma until 7 passages.

### Stability of ADCs and susceptibility to PLM

MMAE (20 µM) and ADCs (equivalent to 20 µM MMAE) were incubated for 0, 0.25, 0.5, 1, 4 and 7 days in 50% human plasma at 37 °C. At the end of the incubation, 1.5 µM PLM was added to determine the susceptibility to PLM. After incubation for 2 h, solutions were prepared as described previously for HPLC. Free MMAE was measured using the liquid chromatography tandem mass spectrometer API3200 LC-MS/MS system (AB SCIEX, USA) as previously described^23^.

### MMAE release from ADCs on IF

Four mg FNG were plated to 48-well culture plate (Corning, USA) and treated with 1 NIU of thrombin solution at 37 °C for 1 h to prepare the IF-coated plates. ADCs (1 µM) were incubated in the uncoated or human.

IF- coated plate with 150 nM PLG, 0.03 nM PA and 100 nM α_2_-AP overnight, the quantities of which were based on the ratio of normal physiological level of each protein in human plasma, and the reaction was stopped by adding three-fold (v/v) acetone. Proteins were removed and reconstituted with acetonitrile. Free MMAE was measured using the liquid chromatography tandem mass spectrometer API3200 LC-MS/MS system (AB SCIEX, USA).

### *In vitro* cytotoxicity assay

One hundred twenty five µg FNG were plated to 96-well culture plate (Corning, USA) and treated with 1 NIU of thrombin solution at 37 °C for 1 h to prepare the IF-coated plates. In 96-well cell culture plates (Corning, USA), 5–11 cells were seeded on the IF-coated plate or non-coated culture plate at 2000 cells/well. On the next day, 0, 0.0125, 0.025, 0.05, 0.125, 0.25, 0.5 and 1.25 µg/mL of Control- or IF-ADC was added to the plate with 1.5 µM PLG, 0.3 nM PA and 1.0 µM α_2_-AP and incubated for 72 h at 37 °C. After incubation, cell viability was assessed with Cell Counting Kit-8 (Dojindo Molecular Technologies, Japan).

### Antitumour effect on xenograft model

Four-week-old BALB/c athymic nude mice (SLC, Japan) were obtained from suppliers and acclimatised for at least 1 week. Five- to 6-week-old mice were transplanted with 5–11 cells at 5 × 10^5^ cells/mouse on the left flank of the back. Administration of each formulation started when the tumour volume reached approximately 200–250 mm^3^ with 20 mg/kg Control- or IF-ADC (equivalent to 0.3 mg/kg MMAE) or 0.3 mg/kg MMAE twice a week, 12 times total intravenously. Dulbecco’s Phosphate-Buffered Saline (D-PBS) was administered as a vehicle control (Vehicle) at equal volume of ADCs (10 µL/g body weight). The tumour volume measurement was performed by digital caliper and calculated as length × breadth^2^/2.

### Evaluation of MMAE distribution in the tumour at timed intervals using LC-MS/MS and IMS

To evaluate the intratumour distribution of free MMAE, BALB/c athymic nude mice bearing 5–11 xenografts were given 20 mg/kg Control- or IF-ADC, and the tumours were resected 1, 6, 24 and 72 h after administration. Tumours were frozen with dry ice and sectioned at 8 μm. MMAE was extracted from the sections with acetonitrile and measured with LC-MS/MS systems as previously described^23^.

In order to visualise free MMAE released from ADCs, the IMS analysis of tumour frozen sections of 5–11 tumours was performed using an atmospheric pressure MALDI-ion trap (IT) time-of-flight (TOF) mass spectrometer (Shimazu, Japan) as previously described^23^.

### Stable supply of KPC mice using *in vitro* fertilization and embryo transfer (IVF-ET)

In the first step, a *Ptf1a-Cre* male mouse^30^ was crossed in its background to a C57BL/6 J female by IVF-ET^27,28^. A total of 270 heterozygous female *Ptf1a-Cre* mice were selected by genotyping. In the next step, a heterozygous *LSL-Kras*^*G12D*^; homozygous *LSL-Trp53*^*R172H*^ double knock-in male mouse^31,32^ (preparation for mating in advance) was crossed to 270 *Ptf1a-Cre* female mice by IVF. Approximately 5,000 fertilised eggs were obtained and frozen until use. The last step for each experiment was to prepare over 20 female KPC mice (all mutant genes [*LSL-Kras*^*G12D*^*/LSL-Trp53*^*R172H*^*/Ptf1a-Cre*] were heterozygous)^25^. Approximately 500 frozen embryos were transplanted in pseudo-pregnant ICR female mice, and approximately 250 mice were born. Over 20 female mice were produced using genotyping by PCR. This procedure was repeated 4 times to obtain totally more than 80 KPC mice.

### Survival of KPC mice receiving ADCs

A stable supply of KPC mice was obtained using the *in vitro* fertilization and IVF-ET method described above. Administration of each formulation was started when mice were reached 8 weeks old with 20 mg/kg ADCs (0.3 mg/kg MMAE, 10 μL/g body weight) twice a week, 12 times total intravenously via lateral tail-veins (n = 20 for all groups). D-PBS was used for vehicle and administered as a control at equal volume of ADCs. The study was approved by the Committees for Animal Experimentation of the National Cancer Centre. All experimental protocols and animal procedures were performed in compliance with the Guidelines for the Care and Use of Experimental Animals established by the Committee for Experimental Animals of the National Cancer Centre. These guidelines meet the ethical standards required by law and comply with the guidelines for the use of experimental animals in Japan.

### Statistical analysis

Data organization and scientific graphing were performed using Microsoft Excel (version 16.0.4549.1000). Statistical analysis was performed using EZR on R commander33 (Version 1.32).

The ELISA data and LC-MS/MS data were evaluated using a two-tailed Dunnett’s test. The last observation carried forward analysis was performed for mice terminated due to tumor size approaching the humane endpoints or sudden death. Tumor growth curves were compared between treatment arms using one-way repeated measures analysis of variance (ANOVA) using 0 to 43 days to avoid missing data considerably affect to analysis results. Survival analysis was undertaken using Kaplan-Meier and the Log-rank test followed by P value adjustment by Holm method. P < 0.05 was considered significant.

## Electronic supplementary material

Supplementary information

## Data Availability

The data supporting the findings of this study are available within the article and its Supplementary Information files and from the corresponding authors on reasonable request.
