# Supplementary material for: Chemotherapy payload of anti-insoluble fibrin antibody-drug conjugate is released specifically upon binding to fibrin
Source: Sci Rep. 2018 Sep 21;8:14211. doi: 10.1038/s41598-018-32601-0 (PMC6155080; doi:10.1038/s41598-018-32601-0)
Supplement: Supplementary file 1 — Supplementary information [file 41598_2018_32601_MOESM1_ESM.pdf]

Chemotherapy payload of anti-insoluble fibrin antibody-drug conjugate is released specifically upon binding to fibrin

Submission category: Article

Authors and affiliations

Hirobumi Fuchigami[1,2], Shino Manabe[3], Masahiro Yasunaga[1,2],

& Yasuhiro Matsumura[1,2*]

[1]Division of Developmental Therapeutics, Exploratory Oncology Research & Clinical Trial Centre, National Cancer Centre, 6-5-1 Kashiwanoha, Kashiwa, Chiba 277-8577, Japan

[2]Department of Integrated Bioscience, Graduate School of Frontier Sciences, The University of Tokyo, 5-1-5 Kashiwanoha, Kashiwa, Chiba 277-8561, Japan

[3]Synthetic Cellular Chemistry Laboratory, RIKEN, 2-1 Hirosawa, Wako, Saitama 351-0198, Japan

*Corresponding author: Yasuhiro Matsumura, yhmatsum@east.ncc.go.jp

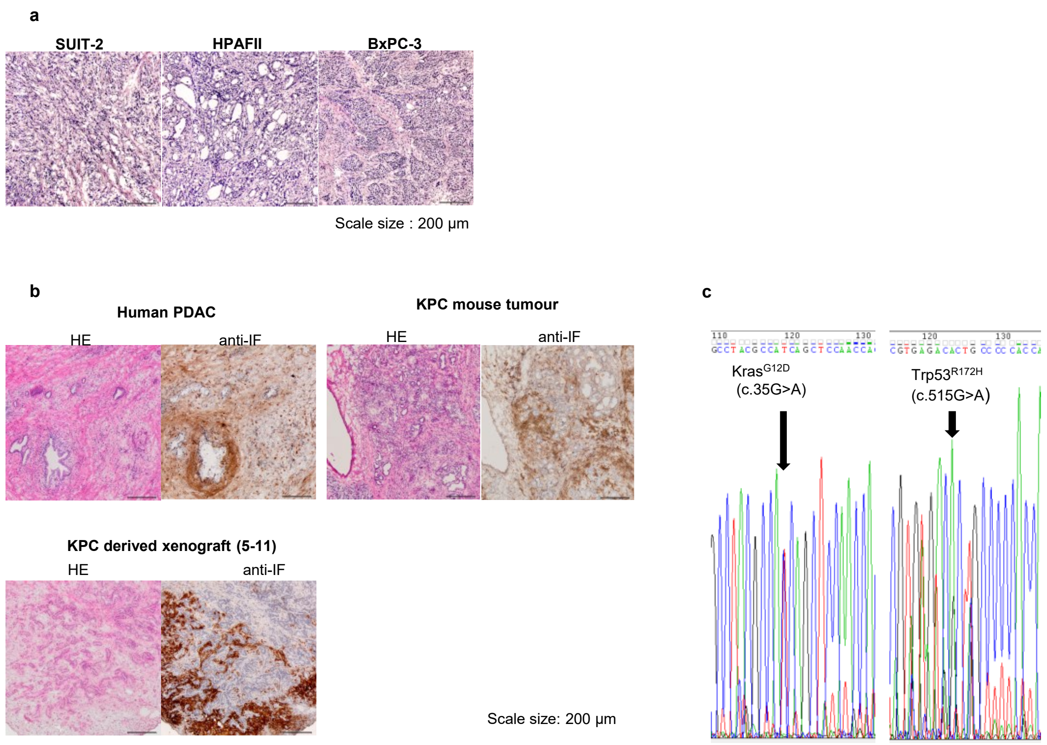

Supplementary Figure 1 Tissue staining of 5-11 tumour, KPC mouse tumour, and human pancreatic ductal adenocarcinoma (PDAC). (a) Hematoxylin-Eosin (HE) staining of the subcutaneous tumour model of various pancreatic cancer cell lines in athymic nude mice. (b) The KPC mouse tumour and 5-11 cell established from a KPC mouse shows a similar property to clinical tumours of human pancreatic cancer. Left panel shows HE staining, right panel shows IHC of anti-IF mAb. (c) Confirmation of KRAS and Trp53 mutation of 5-11 by direct sequencing. Kras^{G12D} mutation and Trp53^{R172H} mutation were maintained in 5-11 cells.

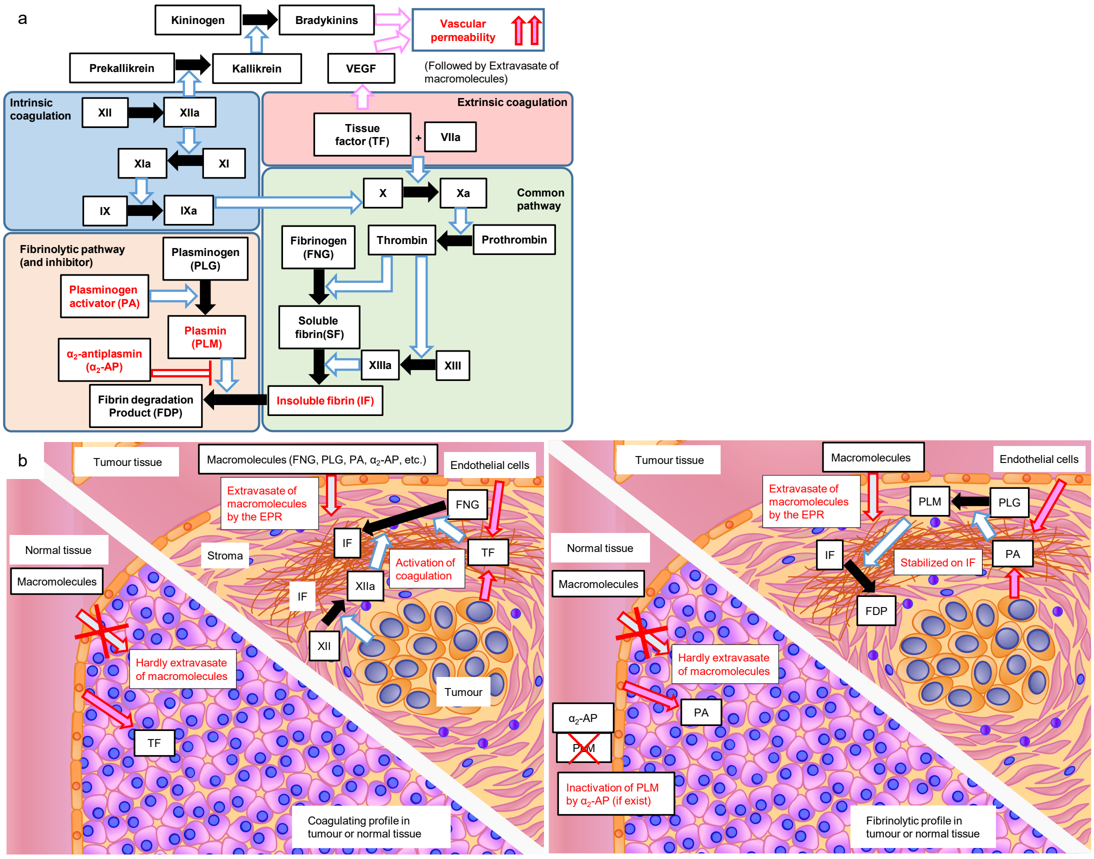

Supplementary Figure 2 Diagram of coagulation and fibrinolysis in normal tissue and tumour tissue. (a) Coagulation and fibrinolysis cascades. Intrinsic or extrinsic coagulation cascades are activated resulting in the formation of IF and enhanced vascular permeability. (b) Coagulation / fibrinolytic profile of normal or tumour tissue. In tumour tissue, high-molecular weight blood coagulation factors extravasated due to enhanced permeability and were activated by TF or factor XIIa resulting in the formation of IF. Fibrinolytic factors also extravasate and are activated on IF. In normal tissue, high-molecular proteins of coagulation/fibrinolysis in blood hardly extravasate.

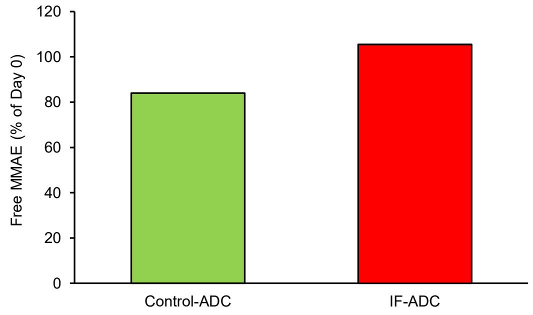

Supplementary Figure 3 Release capability of ADCs after 7 days of incubation in plasma (n=1). Seven days after the incubation in plasma, PLM was added 2 h before the end of incubation. Released MMAE was then measured. The results showed that Control- and IF-ADC could completely release MMAE after the addition of PLM to the plasma.

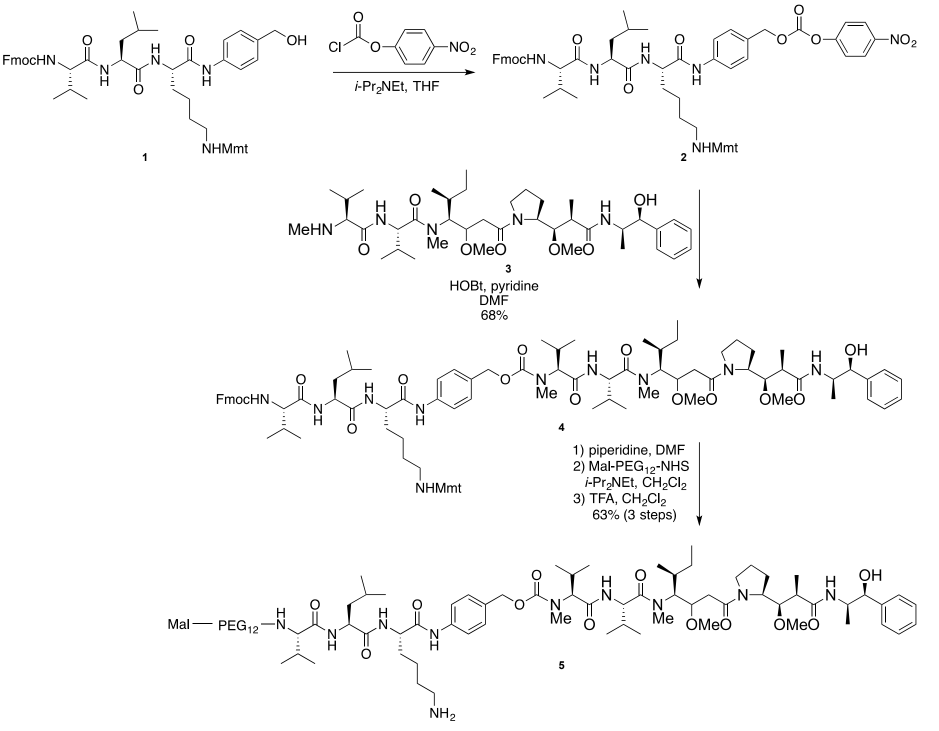

Supplementary Figure 4 The scheme of Mal-PEG12-Val-Leu-Lys -MMAE preparation. The *p*-aminobenzyl carbamate spacer was introduced between the tripeptide Val-Leu-Lys sequence and MMAE. After cleavage of the amide bond between Lys and benzyl alcohol, MMAE is released by the 1,6-elimination reaction. Preparation of Mal-PEG12-Val-Leu-Lys-OPAB-MMAE is as follows; i) preparation of Fmoc-Val-Leu-Lys(Mmt)-OPAB-PNP carbonate **2**; ii) addition of MMAE **3** to Fmoc-Val-Leu-Lys(Mmt)-OPAB-PNP **2** to yield Fmoc-Val-Leu-Lys(Mmt)-OPAB-MMAE **4**; iii) removal of Fmoc group of Fmoc-Val-Leu-Lys(Mmt)-OPAB-MMAE **4** to yield H-Val-Leu-Lys(Mmt)-OPAB-MMAE; iv) addition of Mal-PEG12 to

H-Val-Leu-Lys(Mmt)-OPAB-MMAE to yield Mal-PEG$_{12}$-Val-Leu-Lys(Mmt)-OPAB-MMAE **5**; v) removal of Mmt to yield Mal-PEG$_{12}$-Val-Leu-Lys-OPAB-MMAE.

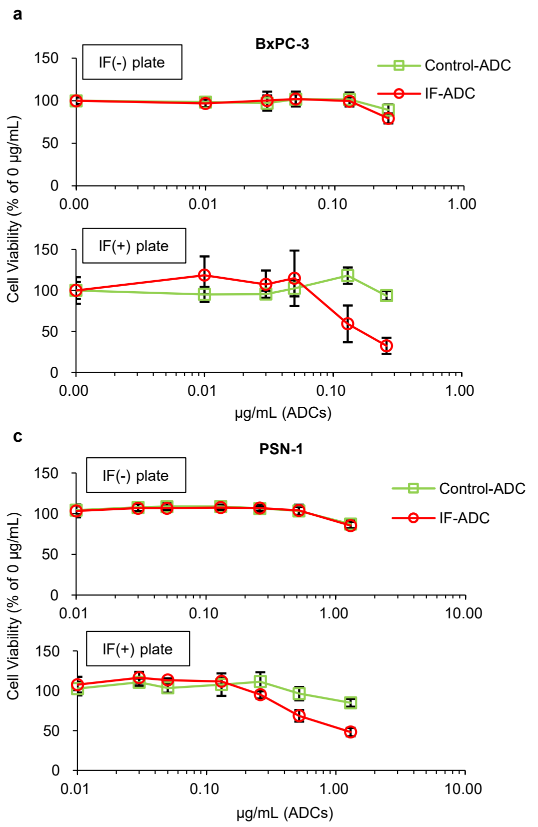

Supplementary Figure 5 *In vitro* cytotoxicity of ADCs using various pancreatic cancer cell lines. Two thousand each cells were incubated with 0-0.26 µg/mL (0.52 µg/mL for PSN-1) ADCs and media containing 0.03 nM PA, 150 nM PNG and 100 nM α_2-AP in the absence/presence of IF (n=4). IF-ADC was only cytotoxic for each cell line on the IF-coated plate at least assayed concentration whereas Control-ADC did not exhibit cytotoxicity regardless of the presence or absence of IF. (a) Cytotoxic of IF-ADC for BxPC-3. The IC_{50} value of IF-ADC was 0.17 µg/mL. (b) Cytotoxic of IF-ADC for PANC-1. The IC_{50} value of IF-ADC was 0.16 µg/mL. (c) Cytotoxic of IF-ADC for PSN-1. The IC_{50} value of IF-ADC was 1.19 µg/mL.

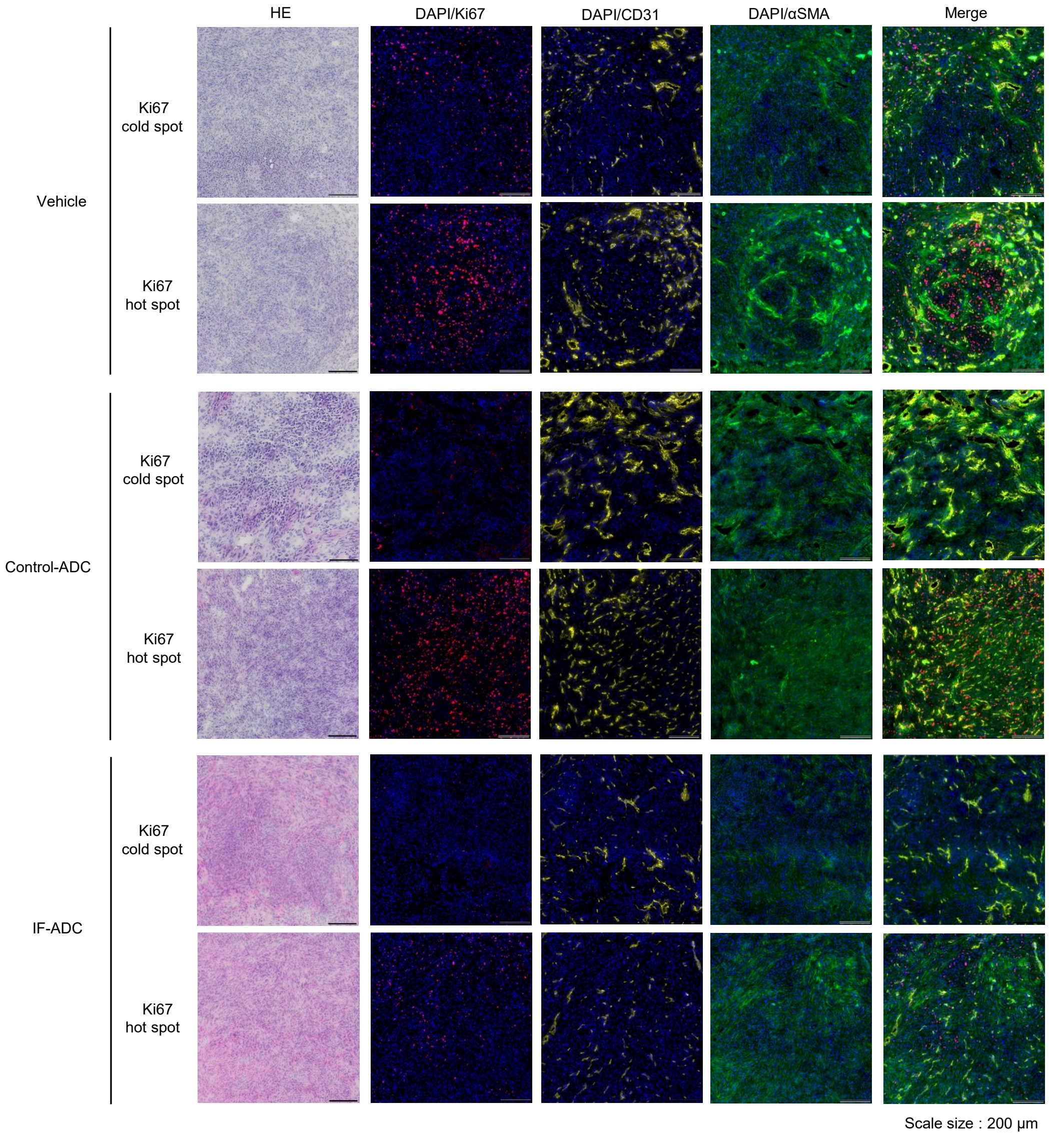

Supplementary Figure 6 Immunohistochemistry of 5-11 subcutaneous tumours. Twelve- to 16-week-old mice were inoculated with 5-11 cells at 5×10^5 cells/mouse on the left flank of the back. Administration of each formulation started when the tumour volume reached approximately 100-150 mm^3 with 20 mg/kg Control- or IF-ADC (equivalent to 0.3 mg/kg MMAE) three times a week, 3 times total intravenously (n=1).

D-PBS was administered as a vehicle control (Vehicle) at equal volume of ADCs (10 μL/g body weight). One day after the last administration, tumours were resected and immunohistochemistry against Ki67, CD31 and αSMA was performed respectively. Data were shown for Ki67 positive hot spot and cold spot, the area with the lowest density of Ki67 positive tumor nuclei.

Supplementary Table 1a Particle size of ADCs.

Compound	Particle size (nm)
Control mAb	13.9 ± 6.4
Control–ADC	16.7 ± 10.0
IF mAb	14.7 ± 5.9
IF–ADC	19.9 ± 9.9

ADC; antibody drug conjugate, IF; insoluble fibrin

Supplementary Table 1b Quantification of free MMAE released from ADCs

Compound	PLM	MMAE (μM)
Vehicle	+	n.d.*
MMAE	+	10.9
Control–ADC	−	n.d.*
	+	10.4
IF–ADC	−	n.d.*
	+	10.7

PLM; plasmin   MMAE;monomethyl auristatin.

*: Not detected

Supplementary Table 2a Stability of MMAE and ADCs in human plasma.

Compound	Time (days)	MMAE (nM)
Vehicle	7	0.0**
MMAE	0	18.5
	0.25	17.5
	0.5	17.8
	1	19.6
	4	19.6
	7	16.7
Control–ADC	0	0.0**
	0.25	0.1**
	0.5	0.1**
	1	0.1**
	4	0.1**
	7	0.2**
IF–ADC	0	0.0**
	0.25	0.1**
	0.5	0.1**
	1	0.1**
	4	0.2**
	7	0.2**

**: Below lower limit of quantification (<1.0 nM)

Supplementary Table 2b. Release capability of ADCs by PLM after 7 days of incubation in human plasma.

Compound	Released MMAE after (nM)
Control–ADC	16.8
IF–ADC	21.1

Supplementary Table 2c Free MMAE from ADCs on IF with Plg, PA and α_2-AP

Compound	IF	MMAE (nM)
Control–ADC	−	0.3 ± 0.0**
	+	1.4 ± 0.4
IF–ADC	−	0.8 ± 0.1**
	+	6.8 ± 0.6

**: Below lower limit of quantification (<1.0 nM)

Supplementary Table 3 Time course of free MMAE in tumour

Compound	Time (h)	MMAE (pmol/g tumour tissue)
Vehicle	1	n.d.*
MMAE	1	97.1
	6	26.9
	24	n.d.
	72	n.d. *
Control–ADC	1	3.3
	6	62.9
	24	22.1
	72	3.8
IF–ADC	1	25.3
	6	49.4
	24	86.0
	72	8.3

*: Not detected

Supplementary Table 4 Percentage of Ki67 positive cells in 5-11 tumour

Compound	Ki67 (counts)	DAPI (counts)	Percentage (Ki67/DAPI)
Vehicle	1384	5876	23.5
Control–ADC	3861	7649	50.5
IF-ADC	555	4754	11.7
